# Prostate cancer risk in men of differing genetic ancestry and approaches to disease screening and management in these groups

**DOI:** 10.1038/s41416-021-01669-3

**Published:** 2021-12-18

**Authors:** Jana McHugh, Edward J. Saunders, Tokhir Dadaev, Eva McGrowder, Elizabeth Bancroft, Zsofia Kote-Jarai, Rosalind Eeles

**Affiliations:** 1 The Institute of Cancer Research, London, UK; 2 The Royal Marsden NHS Foundation Trust, London, UK; 3 Division of Genetics and Epidemiology, The Institute of Cancer Research, London, SM2 5NG UK

**Keywords:** Cancer genomics, Cancer genetics

## Abstract

Prostate cancer is the second most common solid tumour in men worldwide and it is also the most common cancer affecting men of African descent. Prostate cancer incidence and mortality vary across regions and populations. Some of this is explained by a large heritable component of this disease. It has been established that men of African and African Caribbean ethnicity are predisposed to prostate cancer (PrCa) that can have an earlier onset and a more aggressive course, thereby leading to poorer outcomes for patients in this group. Literature searches were carried out using the PubMed, EMBASE and Cochrane Library databases to identify studies associated with PrCa risk and its association with ancestry, screening and management of PrCa. In order to be included, studies were required to be published in English in full-text form. An attractive approach is to identify high-risk groups and develop a targeted screening programme for them as the benefits of population-wide screening in PrCa using prostate-specific antigen (PSA) testing in general population screening have shown evidence of benefit; however, the harms are considered to weigh heavier because screening using PSA testing can lead to over-diagnosis and over-treatment. The aim of targeted screening of higher-risk groups identified by genetic risk stratification is to reduce over-diagnosis and treat those who are most likely to benefit.

## Introduction

**Fig. 1:**
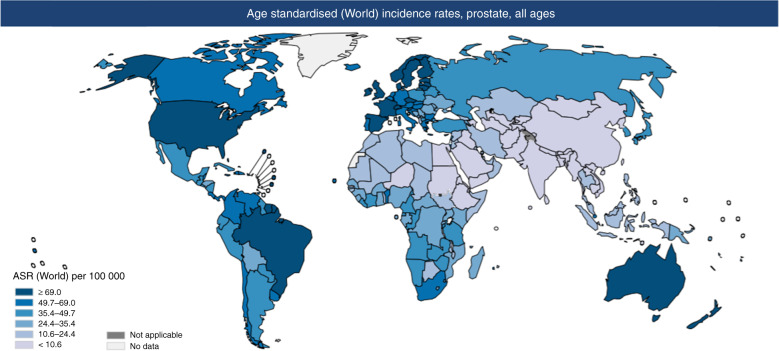
World map showing age standardised world incidence rate of prostate cancer. Map showing estimated age-standardised incidence rates for PrCa worldwide in 2018, in males including all ages [1].

Prostate cancer is common worldwide (Fig. 1). It is the second most common malignancy in men worldwide [1, 2] but not all men will develop a form of PrCa that will be life-limiting.

The use of prostate-specific antigen (PSA) screening alone does not allow us to accurately discriminate between clinically significant disease and disease that will not affect an individual’s overall survival [2, 3].

**Fig. 2:**
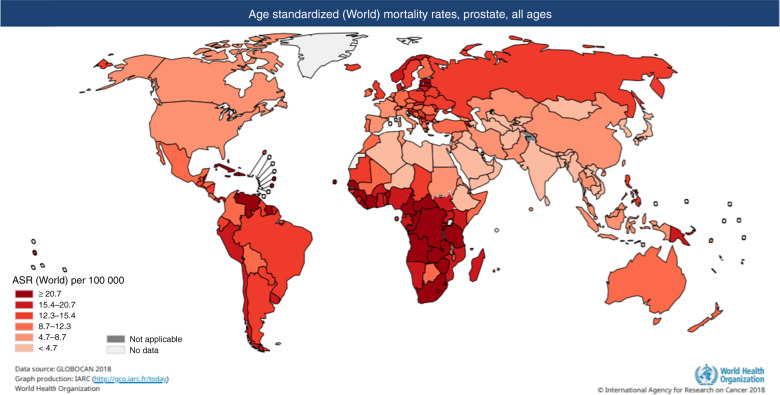
World map showing age standardised world mortality rate of prostate cancer. Map showing estimated age-standardised mortality rates for PrCa worldwide in 2018, in males including all ages [1].

As seen in Figs. 1 and 2 above and below, there are variations in incidence and mortality across countries. The difference in incidence throughout the world may partly be explained by the differences in diagnostic testing and use of PSA screening in some countries, as well as under-reporting and lack of cancer registries, particularly in some developing countries. There are several causes for the variation in mortality, including genetic risk and also the role of the environment [4]. Global mortality differences also likely reflect less access to early detection and certain therapies in the developing world [5].

However, there is evidence demonstrating significant differences in mortality rates depending on ancestry; the African Caribbean population have the highest rates in the world (26.3 per 100,000), whereas men of Asian ancestry have the lowest (2.5 per 100,000) [6].

It has been shown that men of African and African Caribbean ancestry are at higher risk of developing a more aggressive form of PrCa and of developing it at a younger age [7, 8] when compared with men of European ancestry. Therefore, this is a group where ideally we want to establish robust screening tools to improve survival, by detecting clinically significant disease earlier and treating it appropriately. The causes of increased incidence and mortality of PrCa in men of African ancestry are very complex and there are socioeconomic and cultural factors as well as genetic factors contributing to this. Early studies in sub-Saharan Africa highlight these. In some parts of these regions, less than 2% of cases are operable [9].

Genome-wide association studies (GWAS) have identified over 170 common risk alleles for PrCa [10, 11], including the susceptibility region on chromosome 8q24. This region harbours multiple variants that have been suggested to contribute to ethnic differences in PrCa risk [12–14]. We can identify these susceptibility variants, usually single nucleotide polymorphisms (SNPs), which are thought to contribute to an increased risk of PrCa when compared with the average population. GWAS involving people of non-European ancestry such as African ancestry populations including African Americans and peoples of the Caribbean, as well as continental African populations, have been shown to lag behind in the initial GWAS publications, and there is a potential benefit of research with more diverse non-European populations in understanding health disparities worldwide [15].

## Prostate cancer in populations of differing heritage

Ethnicity has many facets, is multifactorial, and can change over time; various possible ways of measuring ethnic groups are available and are used in different countries throughout the world. These include, but are not limited to, country of birth, nationality, language spoken at home, heritage, national/geographical origin and religion. It appears to be generally accepted that ethnicity includes all these aspects, and others, in combination. In the United Kingdom (UK), ethnicity is categorised broadly into categories White, Black, Asian, Mixed and Other ethnic groups and based on the 2011 census data, 13% of the British population belong to a Black, Asian, Mixed or other Ethnic groups (BAME) [16]. However, in this review, we will not use BAME as a category but will discuss the various groups individually.

In the United States of America (USA), commonly used categories used for ethnicity include; Non-Hispanic White, Non-Hispanic Black, Asian/Pacific Islander, American Indian/Alaskan Native and Hispanic [17], and race is used for the classification of continental geographic ancestry.

Retrospective studies from the USA and African countries report a higher occurrence of PrCa in men of African ancestry when compared with men of other ancestries [18]. In the UK, a study was carried out which broke down the lifetime risk of being diagnosed with, and dying from, PrCa in England by major ethnic group: men of Asian ancestry, European ancestry and African ancestry. This did support that men of African ancestry (AFR) are at double the lifetime risk of being diagnosed with PrCa in England, compared to men of European ancestry (EUR) [19]. Another UK-based study looked at males residing in four areas: Bristol, South-West London, South-East London and North-East London [20, 21]. This study also found that AFR men in the UK are at greater risk of developing PrCa compared with EUR men. However, the risk of developing PrCa for men of African ancestry in the UK was slightly lower than the risk in men of African ancestry living in the USA. In the UK men of African ancestry were also found to be more likely to develop PrCa at an earlier age [21, 22].

In the USA, it has been established that African American men are at higher risk of developing PrCa and developing a more aggressive form at a younger age [20, 23]. Asian Americans (this included men of Chinese, Japanese, Filipino, Hawaiian, Korean, Vietnamese, Asian Indian/Pakistani, Pacific islander and other Asian ethnicities), have been found to be more likely to present with more advanced PrCa [24].

Data from an Asian meta-analysis recently published have revealed that although the incidence of PrCa appears lower in Asian countries, the survival rate in countries including Korea, China, Japan, Thailand and India is lower than the survival rates in Europe and North America [25]. In Japan, Japanese PrCa patients were stratified by polygenic risk using 82 SNPs, which were significantly associated with PrCa risk and found that PrCa of earlier onset and cases with a family history of PrCa were enriched in the genetically high-risk population [26].

Overall, there is a paucity of data regarding PrCa in groups of ancestry other than European. It has been noted that men of differing heritage—other than European ancestry, including American African ancestry men (AAM) as well as American men of Hispanic and Asian ancestry are consistently under-enrolled in all trial types for PrCa [27].

There are several factors that need to be discussed when considering cancer health in particular when focusing on particular groups as there are social determinants of cancer health as well as genetic predisposition. Mediators of disease risk can include social factors which may hinder access to healthcare i.e. lower economic standing, lower levels of medical literacy, mistrust of medical services, insurance status and income inequality. Other mediators of disease risk include fitness levels and exercise [28] and obesity and diet [29]. A 2016 review found that men of African ancestry are less likely to seek treatment for PrCa when compared with American men of European ancestry directly or indirectly due to a lack of health insurance or financial barriers [30].

## Prostate cancer in men of African ancestry

A large proportion of the highest age-standardised rate for mortality for PrCa is found in western and southern Africa and in regions of the world with large populations with African ancestry, in particular the Caribbean [8]. It has been difficult to determine the burden of PrCa within continental Africa due to data gaps compounded by a lack of unified systems in PrCa reporting and monitoring [31]. A meta-analysis published in 2016 identified only 40 studies that met the inclusion criteria in 16 countries out of the 54 countries, nine territories and two sovereign states of the African continent, which showed regional incidence rates of PrCa varied widely. It included three studies from Central Africa, two studies from Eastern Africa, four studies from Northern Africa, nine from Southern Africa and 22 from Western Africa [32]. The 2009 literature review by Odedina et al. suggested that the basis of the higher burden of PrCa in American men of African Ancestry (AAM) can be explained in part to the predominantly West and West-central African peoples displaced by the transatlantic slave trade. There is a disproportionate burden of PrCa in men of West African ancestry seen in the UK and in the Caribbean islands [33].

In the USA, the Surveillance, Epidemiology, and End Results (SEER) Program registry data have indicated that AAM tend to be diagnosed at a younger age when compared with men of other ancestries [34]. It is thought that genetic variation could be responsible for part of this effect [35].

It has been noted that men of African ancestry in the USA appear to present with higher stage and more aggressive disease when compared with other ethnic groups [36]. This is thought to be one of the reasons for the annual death rate from PrCa being 2.4 times that of males of European origin [37]. Recent data suggest that West African men also have elevated risk for PrCa compared to European men. Genetic susceptibility to PrCa could account for part of this difference [38]. AAM have a higher PrCa mortality rate and less favourable outcomes when compared with patients of European ancestry [39, 40].

A recent study carried out using two microsimulation models of PrCa calibrated to incidence from the SEER program among AAM projected that different screening strategies (varying screening intervals, starting and stopping ages, and triggering a biopsy following an abnormal PSA) would impact both disease-specific mortality and over-diagnosis. The microsimulation models predicted that screening AAM aged 40–84 years annually would increase both mortality reduction (29–31%) and over-diagnosis (112–129 per 1000). Restricting screening to age 45–69 years would still achieve substantial mortality reduction (26–29%) with lower over-diagnosis (51–61 per 1000). Increasing biopsy utilisation to 100% of abnormal tests would further reduce mortality, but substantially increase over-diagnosis [41].

A study carried out at John Hopkins University looked at a cohort of men who met the institute’s criteria for low-risk PrCa and could opt for active surveillance or prostatectomy. The study found that although they met the low-risk criteria, AAM who underwent immediate surgery, had higher rates of adverse pathology when compared with American men of European Ancestry (AEUR). The results were both statistically and clinically substantial; with higher grade and/or higher stage and higher risk of biochemical recurrence seen in men of African ancestry [42].

In a recently published American study, which sought to examine the association of African American race with conservative management with active surveillance or watchful waiting in the Veterans Health Administration (VA), i.e. a large equal-access health system, it was noted that conservative management was less commonly used and less durable for African American veterans than for White veterans [43]. The authors suggest that prospective trials should be designed to assess the comparative effectiveness of conservative management in African American men with prostate cancer. This may help to answer how best to manage these men.

In the USA, studies have found that even taking into account certain confounding factors such as potentially unequal access to medical care and early detection of cancer, PrCa can progress more quickly in AAM with triple the rate of distant metastasis when compared with AEUR men [44, 45]. A study carried out on 3173 men in the USA between 1994 and 1995 revealed that clinically significant PrCas were detected more frequently in AAM (12.3%) and men of Hispanic ancestry (10.5%) than in AEUR men (6.3%). The risk of clinically advanced stage PrCa remained statistically significantly increased for AAM but not for men of Hispanic ancestry when covariates including socioeconomic factors were adjusted [39].

There have also been some emerging data that in the metastatic setting AAM have better overall survival when using newer hormonal therapies (abiraterone and enzalutamide) when compared with AEUR men [46]. It is recognised that further trials are needed to validate this and explore the mechanisms of racial disparities in outcomes with new hormonal agents. A study reported in 2019, has found that in contrast with evidence indicating worse outcomes for AAM with PrCa at the population level, AAM with metastatic PrCa may respond better to systemic therapy compared with men of non-African ancestry in the USA [47]. This study looked at over 8000 men with metastatic castrate-resistant PrCa enrolled across nine phase III trials where men received docetaxel or docetaxel-containing regimens, and when controlled for known prognostic variables, AAM had better survival outcomes compared with non-AAM patients.

These studies highlight that PrCa incidence and mortality differs across population groups. It is noted in most papers addressing the apparent racial disparity in PrCa outcomes that there is a need to have adequate representation of AFR men and men in other high-risk groups in research on PrCa screening, as well as risk assessment and treatment as these men may gain most from improved screening and care. Research is ongoing in the African continent and although this has its challenges, researchers feel it holds untapped potential to add to the current understanding of the global issue of PrCa, as the second most common cancer to affect men worldwide [9].

## Screening for prostate cancer

The question of whether developing population screening for PrCa should be undertaken, remains unresolved, unlike breast cancer screening population programmes in women. Although PSA testing has been used in several countries for PrCa screening, it has been proven to have limitations [3, 48]. In the past 20 years, PSA screening has been shown to have some downsides that include the under-screening of certain men, in particular younger men, the over-screening of older men and the over-treatment of low-risk disease [48]. The financial impact, the risk of over-diagnosis and over-treatment are the main obstacles to the implementation of population screening programmes solely based on PSA testing [2].

A study looking at the risk of PrCa in men of Caribbean and African ancestry in the UK has suggested that there is a common genetic aetiology [20]. It would, therefore, be logical to consider that in certain higher-risk groups i.e. with higher incidence rates of significant PrCa, such as in men with African and African Caribbean ancestry, the development of a targeted screening programme utilising PSA and other tools including genetic polygenic risk scores may be justified [49].

The screening for PrCa varies across the world and has continued to evolve with guidelines changing over time. In the USA in 2012, the United States Preventive Services Task Force (USPSTF) recommended against individualised PSA screening, due to the emerging evidence of risk of false-positive findings, over-diagnosis and potentially over-treatment outweighing the benefits at the population level. This led to some concerns in particular regarding AAM, as outlined by McGinley et al., that the recommendation against PSA-based screening could have a negative effect on PrCa detection and diagnosis in AAM and worsen the PrCa disparity [23]. This was reviewed by Mahal et al. and it raised the possibility that PrCa outcome among AAM was significantly worse in PSA-screening eligible populations and so a recommendation was made that African ancestry should be addressed in further PSA-screening guidelines [50]. This was then taken into account in the 2018 USPSTF recommendation, which identified AAM and men with a family history of PrCa as having a higher risk of PrCa and so stated that they should be supported in making informed decisions about screening i.e. discussing the pros and cons of PSA screening with their physician [51]. Currently, in the UK, there is no population-based screening for PrCa.

A psychosocial aspect to screening must also be taken into account as men are less likely to partake in preventative healthcare when compared with women. In men of differing backgrounds and heritage, it has been found in studies that they may be less likely to partake in PrCa screening due to beliefs surrounding digital rectal examinations and fears surrounding incontinence and impotence should they need treatment, if cancer were detected [52]. Studies have shown there is a need to develop appropriate culturally sensitive patient education about screening to ensure good uptake across all populations [53, 54].

It is now timely to assess the effect of addition of genetic risk scoring to help guide targeted screening for men with higher incidence rates of significant PrCa, such as men of African and African Caribbean ancestry [49].

## Targeted screening for prostate cancer in the higher-risk groups

There is currently no standard screening practice either population-based or individualised, in the UK for PrCa. PSA testing and digital rectal exam have been used to screen for PrCa, but this remains a source of controversy as well as uncertainty and has seen changes in practice over time, worldwide. Screening with serum PSA has been shown to have some limitations when used in population screening given its low specificity [55–59].

In the USA the USPSTF recommended against PSA testing for screening for PrCa in men of any age in 2012 and this was subsequently updated in 2018 as outlined above. The National Comprehensive Cancer Network (NCCN) advise individualised screening choices for high-risk men, specifically including AAM. The American Cancer Society (ACS) recommends that high-risk men, principally AAM and men with one or more first-degree relatives with PrCa should have an opportunity to make an informed decision with their healthcare provider about whether to be screened for PrCa from the age of 45. They should receive information about the uncertainties, risks, and potential benefits associated with PrCa screening as part of the informed decision [60].

The 2013 American Urological Association (AUA) Prostate Cancer Guidelines strongly recommended shared decision-making for men age 55–69 years at intermediate risk, who are considering PSA testing. The AUA panel did not recommend routine PSA testing in men between ages 40 and 54 years at average risk. The expert panel also commented that “for men younger than age 55 years at higher risk (e.g. positive family history or of African ancestry), decisions regarding PrCa screening should be individualised and discussed with their doctor.” This statement was based on results from the updated European Randomised Study of testing for Prostate Cancer (ERSPC); this trial included European men only. The AUA guideline did not provide a clear direction or guidelines for AAM. This also shows the need to develop screening studies as we have not been able to adequately evaluate the benefit of PSA testing of AAM younger than 55 years of age [61, 62] despite the evidence showing they are at higher risk of developing PrCa at a younger age.

As PSA is an imperfect screening tool and the basis for men of African ancestry being at higher risk of PrCa is fundamentally genetic, combining currently available screening tools such as PSA testing with genetic risk profiling may have the potential to inform screening decisions in men of African ancestry (AFR) [2, 3]. Genetically stratifying higher and lower risk AFR men prior to the screening of higher-risk individuals only, would have the potential to improve the use of available healthcare resources whilst also helping reduce the risks of over-diagnosis that has been associated with the use of PSA testing alone.

**Fig. 3:**
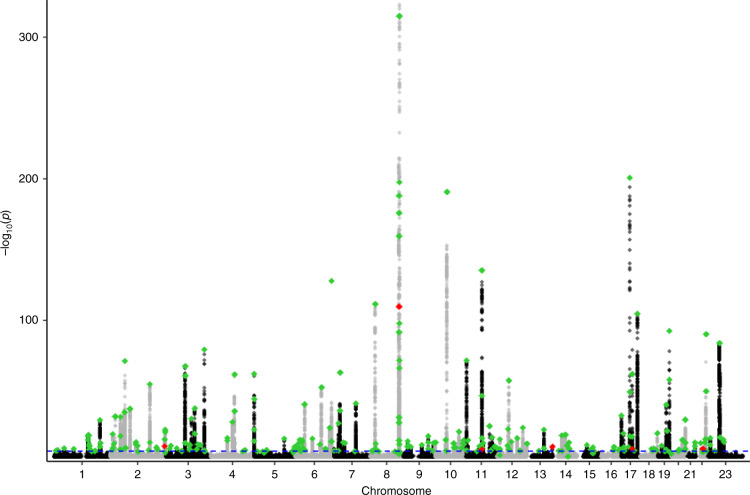
Manhattan plot using the trans-ancestry GWAS January 2021 [65]. The chromosomes are numbered 1–23 in alternating black and grey. The blue dotted line represents genome-wide significance *p* < 5 × 10^−8^. The green dots represent the 269 known PrCa susceptibility loci hits in the multi-ethnicity group. The red dots represent the hits reaching a significant level for AFR but not for EUR. Six regions have specific signals for the AFR population.

The identification of further susceptibility loci and fine-mapping to identify the specific causal variants that drive risk should also help improve the precision of personalised risk prediction and screening decisions so further meta-analyses of international datasets from differing populations are underway. We are aware that diverse populations are largely under-represented in genetics research to date, with studies predominantly examining those of European ancestry [63]. People of non-European ancestry including African, Asian and Hispanic ancestry are generally under-represented in genome-wide studies; however, consortia have been established to identify SNPs that may be of particular importance for AFR men [64]. A trans-ancestry genome-wide association meta-analysis published in Nature Genetics in 2021 [65] identified 269 cross-ancestry PrCa susceptibility loci (Fig. 3), 86 of which are newly discovered risk variants. These included a small number of loci specific to, or substantially enriched in, AFR men, while the inclusion of larger sample sets from AFR men allowed for better refinement of signals within regions and enhanced power for the identification of signals with enriched risk allele frequencies (RAF) among these populations.

**Fig. 4:**
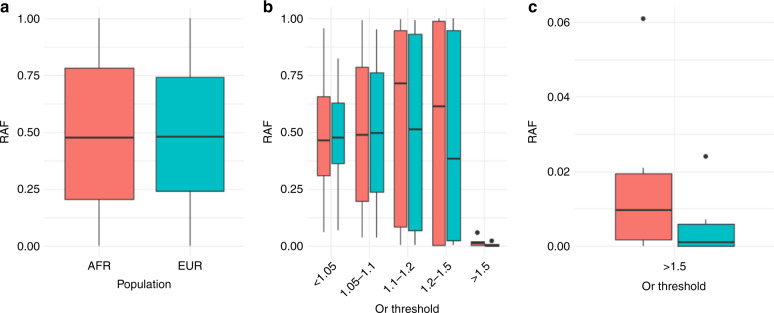
Boxplot depicting risk allele frequency (RAF) of PrCa susceptibility variation in men of African ancestry (AFR) in peach and men of European ancestry (EUR) in cyan. Panel **a** shows the overall RAF in AFR men and EUR men for all 269 variants. Panel **b** Depicts RAFs in both populations stratified by grouped odds ratios from 1 to 1.5, demonstrating that mean RAF of PrCa susceptibility loci is elevated in AFR men for variants with higher effect sizes. **c** An expanded view of RAF for variants with an odds ratio greater than 1.5 showing that these moderate penetrance risk alleles occur more frequently in men of African ancestry.

There are now 269 reported PrCa susceptibility loci and the RAF by population did not substantially differ between EUR men and AFR men, with the average RAF of 0.490 in EUR men and RAF of 0.494 in AFR men (Fig. 4a). When the RAF is stratified by effect size, however, using the odds ratio (OR) grouping by cut-offs from 1 to 1.5, it becomes clear that variants with a higher OR (OR > 1.1) are generally more common in AFR men than in the EUR men and alleles with a lower OR are marginally depleted in AFR men (Fig. 4b). The risk alleles of moderate penetrance risk variants with OR > 1.5, which although less common, have an outsized contribution to PrCa susceptibility and are substantially more enriched in the AFR populations (Fig. 4c). Consequently, the PrCa risk alleles with larger effect sizes that confer a greater risk of PrCa are more common in AFR populations and they are likely to contribute substantially towards the increased rates of PrCa in these ancestral populations. In particular, an African ancestry specific variant has been identified at chromosome 8q24 with OR > 2 with RAF of 6% in the AFR population, and this variant is also significantly associated with a family history of PrCa and younger age of diagnosis in African ancestry populations [66, 67].

These common, predominantly lower penetrance PrCa susceptibility variants enabled the development of polygenic risk scores (PRS), for the prediction of lifetime PrCa risk in individuals. PRS is calculated based on the sum of risk alleles for the PrCa risk loci, weighted by their per-allele log odds ratio.

A large GWAS and replication study on a Japanese cohort (9906 cases and 83,943 male controls) detected 12 novel loci for PrCa, seven of which had very low minor allele frequency in the European population. We recognise that within broad categorisations e.g. Asian population, there are also differences between groups and that a Japanese cohort will not be representative of an overall Asian population. This work is emphasising the fact that in order to accurately identify men at higher risk across diverse populations, it is important to ensure that the PRS is calibrated appropriately for the population being screened and appropriate screening thresholds are established for men of differing genetic ancestries.

**Fig. 5:**
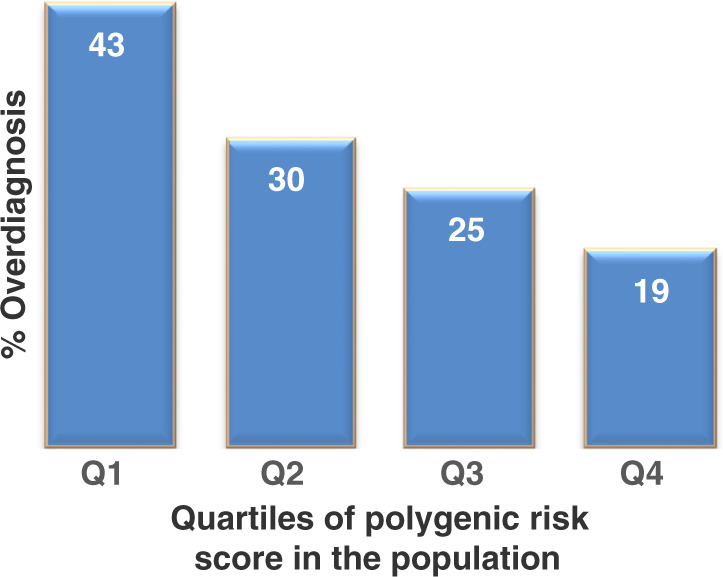
Barchart depicting rate of over-diagnosis of prostate cancer and polygenic risk score in quartiles. Bar chart with rate of over-diagnosis of PrCa by polygenic risk quartile showing the proportion of prostate cancers likely to be over-diagnosed varies inversely with polygenic risk [68].

PRS could therefore potentially be utilised to help target screening specifically towards men with higher risk and it is hoped that these groups will be enriched for developing clinically significant PrCa. Early reports suggest that the use of PRS can help to reduce over-diagnosis of PrCa, and this, in turn, should help reduce the burden on healthcare systems worldwide. In a study carried out looking at the utility of PRS in PrCa and specifically at its use in reducing over-diagnosis, modelling predicts that when the PRS are divided into quartiles (Fig. 5), a lower proportion of over-diagnosed PrCa cases were observed in the highest quartile compared with the lowest quartile [68].

## The PROFILE study

The PROFILE study (NCT02543905) set out to investigate targeted screening in higher-risk men in the UK. The aim of this study has been to investigate the role of targeted PrCa screening in men at a higher genetic risk i.e. men of AFR ancestry or men with a family history of PrCa and its association with specific genetic profiles and biomarkers (both biological samples and imaging). We defined men of African and /or African Caribbean Ancestry as men with all four grandparents of African and/or African Caribbean Ancestry: this was the inclusion criteria for this cohort of the study. The primary endpoint of the study is to determine an association between the genetic profile and the prostate biopsy result. A PSA blood test is done initially and men are offered further screening with MRI of the prostate and prostate biopsy should they be happy to proceed regardless of baseline PSA or offered prostate MRI and biopsy should their PSA be above an age-dependent threshold. We have invited (i) men of African and African Caribbean ancestry and (ii) men of European ancestry with a family history of PrCa to participate in the study.

The pilot phase of the study examined the role of upfront biopsy regardless of PSA in 100 men of European ancestry with a family history of PrCa. A PRS was calculated using known PrCa risk SNPs at that time. In the pilot study, PrCa was detected in 46 of the 136 men who underwent prostate biopsy (33.8%), of which 28.3% (13/46) were clinically significant [69].

We are now recruiting a total of 700 subjects (350/cohort) to investigate the role of targeted screening in men at higher risk for PrCa. Men will be asked to provide a DNA sample which will then be analysed to detect over 130 SNPs [11], with newly identified SNPs being incorporated specifically for the AFR ancestry arm of the study.

## Discussion/conclusions

Screening for PrCa should aim to detect cancers that are clinically significant, and we know that PSA alone remains an imperfect tool to discriminate between clinically significant cancer and cancer that may not affect a man in his lifetime.

It is known that men of African and African Caribbean ancestry, as is seen in men with a known family history of PrCa, are at higher risk than the general population of developing PrCa and potentially developing it at a younger age and with more aggressive disease. Men of African ancestry are an excellent group in which to aim to develop a novel screening programme integrating the latest screening tools of multiparametric MRI and genetic profiling using PRS to identify those at higher risk. The PROFILE study is focusing on men of African and African Caribbean ancestry but this approach will hopefully be able to be applied to other high-risk populations, where using an appropriate PRS to improve screening will be beneficial. The most up to date, emerging PRS will aim to include more SNPs relevant to populations of different ancestries so that one test can be used to stratify populations with mixed ancestry. This study will look at one group of men at higher risk of PrCa with PRS, and in the future should this prove to be beneficial this approach can be utilised in other groups as well.

The overall aim of the PROFILE study is to highlight the utility of using PRS to improve screening and if proves beneficial in the study setting in reducing over-diagnosis and identifying those who are likely to benefit from earlier diagnosis and treatment, with the long term goal being to improve survival. If successful, consideration should be given to including PRS into authoritative guidelines for screening. It will be imperative as our knowledge continues to evolve, that we prioritise ensuring accessibility and availability of PRS-based targeted PrCa screening, if successful in high-risk populations and being inclusive of those with socioeconomic disadvantage and in developing nations.
